# Health care resource utilization and characteristics of patients with eosinophilic asthma in secondary health care in Finland

**DOI:** 10.1080/20018525.2018.1458560

**Published:** 2018-04-15

**Authors:** Mika J. Mäkelä, Helene Nordahl Christensen, Antti Karlsson, Sarang Rastogi, Kirsi Kettunen

**Affiliations:** a Skin and Allergy Hospital, Helsinki University Hospital, and University of Helsinki, Helsinki, Finland; b AstraZeneca, Södertälje, Sweden; c Auria Biobank, University of Turku and Turku University Hospital, Turku, Finland; d AstraZeneca, Gaithersburg, MD, USA; e Medaffcon Oy, Espoo, Finland

**Keywords:** Eosinophil, asthma, resource utilization, medical record data, observational study

## Abstract

**Background**: Eosinophilic airway inflammation is common in asthma patients and appears to be associated with severe exacerbations and loss of asthma control.

**Objective**: To describe the resource utilization and clinical characteristics of patients with eosinophilic asthma.

**Design**: Asthma patients ≥18 years with ≥1 blood eosinophil count in secondary care (South West Finland) during 2003‒2013 were included. Clinical characteristics (age, lung function, body mass index, and comorbidities) and asthma-related resource utilization (hospital admissions, outpatient visits, and emergency room [ER] visits) were retrieved. Resource utilization rates were compared for patients with blood eosinophil ≤ or >300 cells/μL, using adjusted negative binomial regression models.

**Results**: Overall, 4,357 eligible patients were identified (mean age 60 years, females 68%), of which 1,927 (44%) had >300 eosinophil cells/μL blood. Patients with ≤300 and >300 eosinophil counts, exhibited similar clinical characteristics, including advanced age, poor lung function, and overweight. Comorbidities such as pneumonia, sinusitis, and nasal polyps, were more frequent among those with >300 eosinophil cells/μL blood compared with patients with lower counts. Eosinophil counts >300 cells/μL were associated with greater hospital admissions (rate ratio [RR] [95% confidence interval CI]: 1.13 [1.02;1.24]) and outpatient visits (RR [95% CI]: 1.11 [1.03;1.20]) compared with patients with lower eosinophil counts. Rates of ER visits were similar between the patient groups (RR [95% CI]: 0.99 [0.87;1.12]).

**Conclusions**: Hospital admissions and outpatient visits occurred more often for patients with eosinophil counts >300 cells/µL, than for patients with lower eosinophil counts. Routine blood eosinophil screening might be useful to identify patients with an eosinophilic phenotype eligible for more targeted treatments.

## Introduction

Asthma is a serious global health problem, affecting more than 315 million people in all age groups worldwide [1]. In Finland and several European counties, improvements in asthma care during the last two decades have resulted in cost savings at both societal and patient levels, despite the increasing number of asthma patients [2–5]. However, many severe asthma patients are still uncontrolled [6,7], and asthma imposes a high burden on health care systems and on society [1,8].

Approximately 50% of all asthma patients suffer from eosinophilic inflammation [9–12]. Elevated eosinophil counts in blood and sputum is associated with disease severity [13,14]. As early as 1975, Horn et al. [15] observed that increased blood eosinophil counts correlate with increasing airway obstruction in patients undergoing a pulmonary function test. Since then, several observational studies have also revealed that increased blood eosinophils are associated with more severe airflow obstruction [16], more severe exacerbations [17–20], lower odds of achieving asthma control [17–19,21], and increased hospital costs [22,23]. Patients with eosinophilic inflammation generally respond well to inhaled corticosteroid. However, some patients also require oral corticosteroids, which may be associated with adverse events [24–26]. Treatments targeting eosinophils may improve asthma control [27].

The burden caused by eosinophilic asthma in Finland is not clearly described. To potentiate informed decisions on optimized asthma care management in Finland, we sought to better understand the burden of eosinophilic asthma. The objective of this study was to describe the clinical characteristics and asthma-related health care resource utilization of eosinophilic asthma patients in Finnish secondary health care.

## Methods

### Study design and data source

This was a non-interventional study in secondary care of South West Finland hospital district (i.e., inpatient and outpatient hospital care). Data on clinical characteristics (age, lung function, body mass index [BMI], and comorbidities) and asthma-related resource utilization (hospital admissions, outpatient visits, and emergency room [ER] visits) was retrieved from the Auria Biobank (Turku, Finland) Research Database.

### Study population

The study population comprise of adult, physician diagnosed asthma patients (International Classification of Diseases version 10 (ICD-10): J45 and J46) with data available in the Auria Biobank Research Database, and at least one measurement of blood eosinophils between 1 January 2003 and 31 August 2013.

### Asthma-related health care resource utilization

Asthma-related health care resource utilization was defined as the number of hospital admissions and days, emergency room (ER) visits and secondary care outpatient visits with an asthma diagnosis. The date of the first asthma diagnosis during the study period was defined as the index date. All patients were followed from the index date until death or the end of study (31 August 2013).

### Blood eosinophil count

Patients were classified based on their greatest blood eosinophil counts (cells per μL, blood) during the observation period and grouped into ≤300 eosinophil cells/μL and >300 eosinophil cells/μL according to clinical practice. Health care resource utilization was described using increments of eosinophil cutoff of >150, >300, >450, and >600 cells/μL.

### Clinical characteristics

Lung function, BMI and comorbidities (Charlson Comorbidity Index [28]) were defined as the means of measurements during the observation period. The age at the time of the greatest blood eosinophil count was identified to display age distributions linked to eosinophilic asthma onset.

### Sputum eosinophilic subgroups

Patients who had sputum eosinophil sample taken within a three months time-window from the greatest blood eosinophil sample were classified in a semi-quantitative manner based on the visual number of eosinophil cells per sputum strand into three sputum eosinophil subgroups: ‘none’ and ‘moderate,’ and ‘dominant.’ Sputum eosinophil samples were collected, processed, and analyzed by specialized staff at the TYKSlab [29].

### Statistical analyses

Distribution of clinical characteristics was compared between patients ≤300 and >300 eosinophil cells/μL blood. The χ^2^ test was used for categorical variables. Variables measured on the interval or ratio scale were compared with an ANOVA test or a Mann-Whitney *U* test if the distribution were skewed.

Asthma-related health care resource utilization was reported as crude rates per patient-year (i.e., the sum of all admissions [the number of days or visits] of all patients divided by the sum of all follow-up times for all the patients). Furthermore, a negative binomial regression model (for over dispersed outcome data) was used to compare the rates of resource utilization events (hospital admissions, ER visits, and outpatient visits) for eosinophil counts. The negative binomial analyses were adjusted for the following confounders: age, sex, and comorbidity. As a sensitivity analysis, patients (*n* = 56) who had a follow-up period less than 1 month were excluded.

The overall correlation between eosinophil counts in blood and sputum samples was assessed using the Spearman correlation coefficient in the subpopulation of patients who had sputum eosinophil sample.

Analyses were done with Python 3.5, Scipy stats package, and IBM SPSS Statistics version 24.0.0.1. Visualization was done with Seaborn version 0.7.1.

## Results

### Clinical characteristics

**Figure 1.**
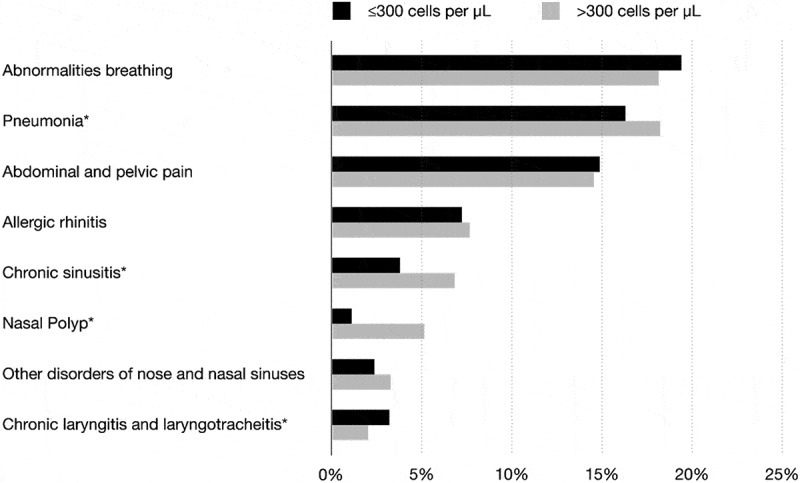
Asthma-related conditions by blood eosinophil count, *p < 0.05.

In total, 7,707 patients with an asthma diagnosis were included (mean age 60 years, females 68%), of which 4,357 (57%) patients had at least one blood eosinophil count (median 270 eosinophil cells/μL blood). A total of 1,927 (44%) patients had blood eosinophil count >300 cells/µL and the majority patients were women (Table 1). Both patient groups (≤ and >300 eosinophil cells/μL) were similar regarding clinical characteristics typically associated with severe asthma, including, for example, advanced age (median age: 60 years), overweight (mean BMI: 28 kg/m^2^), and poor lung function (mean FEV_1_/FVC: 73,80) (*p* > 0.05 for all, Table 1). The most common comorbidity was hypertension (36%) in both patient groups (*p* > 0.05, Table 1). Asthma-related conditions such as pneumonia, sinusitis, and nasal polyp were more frequent among patients with blood eosinophil count >300 cells/µL compared to those with lower counts (*p* < 0.05, Figure 1).

**Table 1.** Patient characteristics, comorbidity and asthma-related health care resource utilization by blood eosinophil count.

		Blood eosinophil level, cells/µL		
	Total	≤300	>300	
	*N = 4357*	*n (%) 2430 (55.77)*	*n (%) 1927 (44.23)*	*p*-value
**Sex**, *n* (%)				0.00
Male	1382 (31.71)	703 (28.93)	679 (35.23)	
Female	2975 (68.28)	1727 (71.07)	1248 (64.76)	
**Age** (years), median (5th–95th percentile)	59.78 (19.31–84.40)	59.54 (20.60–84.41)	60.15 (18.40–84.32)	0.62
**Blood eosinophils** (cells/μL), median (5th–95th percentile)	270.00 (50.00–1040.00)	170.00 (30.00–290.00)	490.0 (320.0–1470.0)	0.00
**BMI** (kg/m^2^), mean (SD)	28.34 (6.21)	28.31 (6.24)	28.37 (6.17)	0.77
Missing Data	1594	900	694	
**Spirometry**, mean (SD)				
Prebronchodilator FEV_1_ (L)	2.34 (0.87)	2.31 (0.86)	2.36 (0.87)	0.13
FEV_1_/FVC Prebronchodilator	73.80 (10.77)	74.09 (10.91)	73.45 (10.59)	0.09
Missing Data	2014	1155	859	
**Comorbidity^a^**, *n* (%)				
Hypertension	1567 (35.97)	865 (35.60)	702 (36.43)	0.59
Arterial fibrillation	642 (14.73)	399 (16.42)	243 (12.61)	0.00
COPD	637 (14.62)	376 (15.47)	261 (13.54)	0.08
Type 2 diabetes	629 (14.44)	351 (14.44)	278 (14.43)	0.98
Sleep disorders	611 (14.02)	330 (13.58)	281 (14.58)	0.37
**Asthma-related health care resource utilization**, n (rate per person year)				
Hospital admissions	6076 (0.29)	3124 (0.28)	2952 (0.31)	0.02*
Outpatient visits	22 728 (1.09)	11 395 (1.01)	11 333 (1.18)	0.01*
Emergency room visits	2265 (0.11)	1232 (0.11)	1033 (0.11)	0.58*
**Patient years**, n	20 819	11 241	9578	

^a^The five most frequent comorbidities.

*p-values adjusted for age and gender.

### Asthma-related health care resource utilization

During the observation period, covering a total of 20,819 patient-years, the main driver of the asthma-related health care resource utilization was outpatient visits with a rate of 1.09 visits per patient-year, followed by hospital admissions (0.29 admissions per patient-year) and emergency room visits (0.11 visits per patient-year) (Table 1). The average length of hospital admissions was 1.50 days per patient-year.

**Figure 2.**
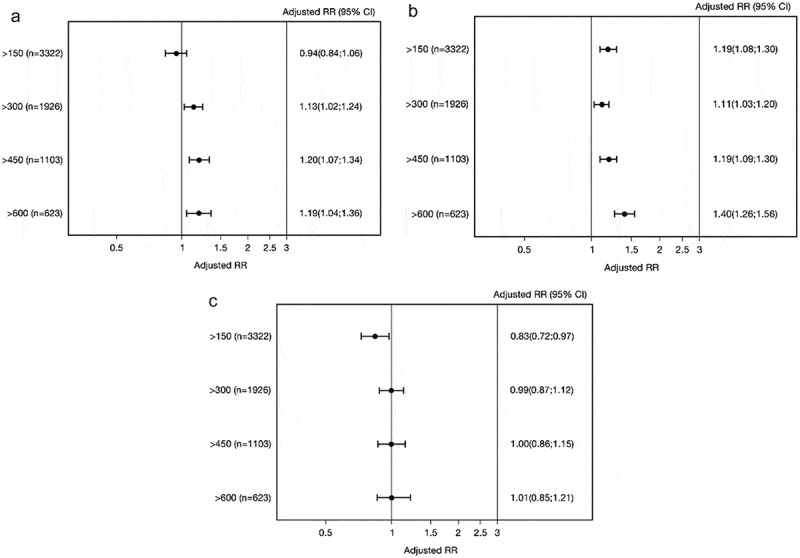
Adjusted rate ratios (RRs) for asthma-related hospital admissions (a), outpatient visits (b), and ER visits (c) for patients at different blood eosinophil cutoff values. Adjusted for age, sex, and comorbidity.

The adjusted rate ratios (RR) were slightly greater for patients with eosinophil count >300 cells/µL for hospital admissions (RR [95% confidence interval CI]: 1.13 [1.02;1.24]) and outpatient visits (RR [95% CI]: 1.11 [1.03;1.20]) compared to those with lower count (Figure 2(a,b)). Among those with >600 eosinophil cells/μL blood (*n* = 623 [14%]) the RR was 1.19 (95% CI: 1.04;1.36) for hospital admissions and 1.40 (95% CI: 1.26;1.56) for outpatient visits (Figure 2(a,b)) compared with those with lower eosinophil count. No differences were observed on emergency room visits (Figure 2(c)).

The sensitivity analysis indicated that excluding patients with less than 1 month follow up did not alter the results (data not presented).

### Subpopulation with sputum eosinophil sample

**Figure 3.**
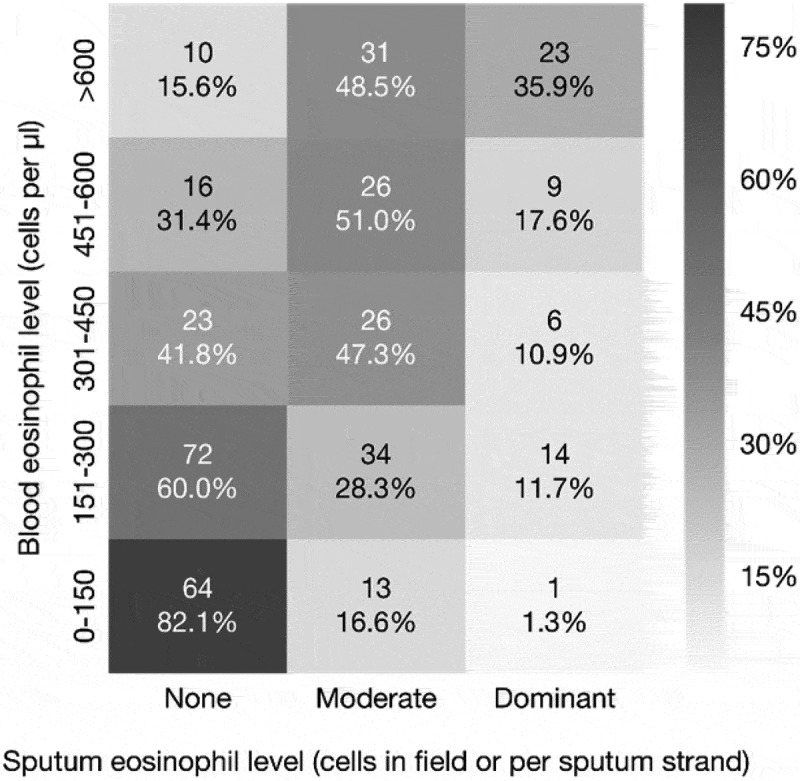
Correlation between eosinophil samples from blood and sputum (the Spearman correlation coefficient 0.49, *p* < 0.01).

The analysis of 368 patients (8% of the study population) who had sputum eosinophil sample taken within a 3 months’ time-window from the blood eosinophil sample (mean: 10.5 days [SD: 20.7]) suggested a moderate Spearman correlation coefficient of 0.49 (*p* < 0.01) between blood and sputum eosinophil count (Figure 3).

There was no difference in the adjusted rate ratios (RR) of hospital admissions and outpatient visits for the moderate and dominant sputum eosinophil groups compared to the non-sputum eosinophil group (Table 2). However, for patients with dominant sputum eosinophils, the RR of emergency room visits was 3.10 (95% CI: 1.33;7,24) compared with patients in the non-sputum eosinophil group (Table 2).

**Table 2.** Adjusted rate ratios (RRs) for asthma-related hospital admissions, outpatient visits, ER visits for patients at different sputum eosinophil subgroups.

	Hospital admissions		Outpatient visits		ER visits	
Sputum eosinophils	Events	RR (95% CI)^a^	Events	RR (95% CI)^a^	Events	RR (95% CI)^a^
None (*n* = 185)	104	Ref	1040	Ref	23	Ref
Moderate (*n* = 130)	64	0.86 (0.55;1.36)	885	1.11 (0.89;1.38)	20	1.61 (0.74;3.48)
Dominant (*n* = 53)	44	1.51 (0.88;2.60)	333	0.98 (0.73;1.31)	22	3.05 (1.28;7.27)

^a^rate ratio (95% confidence interval) adjusted for age, sex, comorbidity.

Ref: reference.

## Discussion

Of more than 4,300 adult asthma patients who had a recorded blood eosinophil count in secondary care, more than 40% had a blood eosinophil count of >300 cells/μL. The rate of hospital admission was 13% greater and the rate of outpatients’ visits was 11% greater, for these patients compared with the 300 cells/μL or less eosinophil group. These findings correspond to the findings of other observational studies [21–23] and support that routine blood eosinophil screening practices might be of value for identification of patients with elevated eosinophil for more targeted treatment plans. A recent meta-analysis of 20 studies involving 7,100 asthma patients found anti-IL-5 therapies with different mode of actions could provide clinical benefit for patients with severe, eosinophilic asthma [27].

Traditionally, management of asthma patients has been based on assessment of symptoms, lung function, and use of rescue medication [30]. However, assessing the underlying eosinophilic airway inflammation may add an extra feature to asthma management [31]. In our study, more than half of the patients had at least one blood eosinophil count whereas less than 10% of the patients had a sputum eosinophil count. This might reflect that the blood eosinophil count is an attractive biomarker because of the ease of availability and its potential to identify patients with asthma eligible for treatment with novel therapeutics. In contrast, the measurement of sputum eosinophils is time-consuming and requires specific technical expertise not generally available in clinical settings [32]. Nonetheless, sputum eosinophil counts have been helpful in characterizing airway inflammation, predicting response to corticosteroid treatment, and identifying patients at risk of exacerbations [33–35].

Comorbid upper airway disease is common in patients with asthma [36,37], and has been associated with poor asthma control [38], unscheduled care [39], and impaired health related quality of life [40]. In our study, conditions such as pneumonia, sinusitis, and nasal polyp were more frequent among those with blood eosinophil >300 cells/μL compared to those with lower counts, while other comorbidities did not differ between the groups.

### Limitations

This study has several limitations, and the findings should be interpreted with caution. We used the greatest blood eosinophil count during the study period as a proxy of eosinophil activation, which may have introduced non-differential misclassification leading to a potential attenuation of the results and in such a minor difference between the two groups (≤ and >300 cells/μL). Measures of blood eosinophil count tend to have a great deal of variability [41–43]. Mathur and colleagues [42] found that a typical eosinophil count in any given individual was approximately 40% greater or lesser than that individual’s mean. They also found that only 2% of the within-patient variability in eosinophil counts could be attributed to the month of collection (seasonal allergies), and less than 1% could be attributed to inhaled corticosteroid use. Therefore, repeated measurements of the blood eosinophil count may be necessary to definitively identify the patient’s eosinophil activation level.

In addition, our study may have been prone to selection bias. The study population was identified in the Auria Biobank Research Database, which include patients having a tissue sample taken while being treated in secondary care in the South West Finland hospital district. However, most of the asthma patients typically are treated in primary care. Other observational studies investigating the relation between blood eosinophil count as a biomarker of eosinophils activation and asthma outcomes included patients from primary care [16–19,21–23]. In these studies, the patients were younger and the percentage of patients with blood eosinophil count >300 cells/μL were lower than our patients (between 20‒30%). Thus, our study population would be expected to be an older, more severe or more difficult-to-treat group of asthma patients.

Information on important confounding factors such as treatment and asthma control which could affect eosinophil level and disease progression [44] is lacking in the current study. We cannot rule out that, for some patients, the low eosinophil count was a result of well-controlled use of corticosteroids. However, Casciano and colleagues [23] found that eosinophil elevation leads to greater health care resource utilization and costs, irrespective of control classification. Thus, we tend to believe that our study might give a conservative estimation of the association between blood eosinophil levels and health care resource utilization.

### Generalizability

Almost half of the patients identified initially with asthma (4,357 of 7,707) did not have any measure of blood eosinophil. Patients who have at least one measure of blood eosinophil may be different in clinical presentation or severity (more severe) compared with patients who never have an eosinophil test measured. Therefore, our results may not be generalized to the entire asthma population.

## Conclusions

Health care hospital admissions and outpatient visits were more often observed for patients with eosinophil counts >300 cells/µL blood than for patients with lower eosinophil counts. Routine blood eosinophil screening might be useful to identify patients with an eosinophilic phenotype for more targeted treatments, but also monitor and prevent disease activity in order to obtain disease control and reduce the utilization of health care resources.
